# Do *Abies religiosa* Seedlings That Survived Hot/Dry Range‐Edge Conditions Exhibit Genetic Evidence of Selection?

**DOI:** 10.1002/ece3.74455

**Published:** 2026-10-20

**Authors:** Yinyue Du, Emily V. Moran, Ruli Zhang, Ana Laura Cruzado‐Vargas, Cuauhtémoc Sáenz‐Romero

**Affiliations:** ^1^ Department of Life and Environmental Sciences University of California, Merced Merced California USA; ^2^ College of Forestry Southwest Forestry University Kunming China; ^3^ Instituto de Investigaciones Agropecuarias y Forestales Universidad Michoacana de San Nicolás de Hidalgo (UMSNH) Morelia Michoacán México; ^4^ Instituto de Investigaciones en Ecosistemas y Sustentabilidad Universidad Nacional Autónoma de México (UNAM) Morelia Michoacán México; ^5^ Instituto de Investigaciones Sobre los Recursos Naturales UMSNH Morelia Michoacán México; ^6^ Laboratorio Nacional de Biología del Cambio Climático (LaNBioCC), Secretaría de Ciencia, Humanidades Tecnología e Innovación (SECIHTI), Instituto de Biología, UNAM Ciudad de México Mexico

**Keywords:** *Abies religiosa*, common garden experiment, drought response, forest conservation, GEA, local adaptation, PCAdapt

## Abstract

Sacred fir (*Abies religiosa)* is the only roosting tree for monarch butterfly populations overwintering in Mexico, but its geographic range is expected to shrink due to climate change. A common garden experiment carried out 2019–2022 showed high seedling survival at 3400 and 3000 m, but only 6% survival at 2600 m (representing possible warmer and drier future conditions). We tested whether these survivors were genetically different from the starting population (represented by the seedlings from high‐survival gardens) using PCAdapt. We also conducted a Genotype‐Environment Association (GEA) test using climate information from source populations to identify SNP variants associated with climate gradients. PCAdapt identified 2202 outlier SNPs whose allele frequencies may have been impacted by selection. LFMM_2_ identified 33 SNPs associated with 5 source population climate gradients, especially winter precipitation. Minor allele frequencies (MAF) in both categories showed more fluctuation in survivors. This suggests that local climate conditions may select for genetic differences in *Abies religiosa* on short as well as long timescales, but fully understanding these responses requires more complete genome or transcriptome data, as well as validation by further field tests.

## Graphical abstract

In a common garden experiment of sacred fir (Abies religiosa), nearly all seedlings planted at 3400 and 3000 m survived, but only 6% survival at 2600 m (representing possible warmer and drier future conditions). When comparing low elevation survivors to higher elevation seedlings representing the initial population, PCAdapt identified 2202 outlier SNPs whose allele frequencies may have been impacted by selection; LFMM2 identified 33 SNPs associated with 5 source population climate gradients, especially winter precipitation. This suggests that local climate conditions may select for genetic differences in *Abies religiosa* on short as well as long timescales.

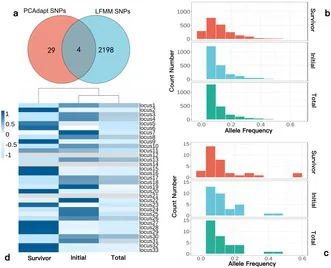

## 1 Introduction

The sacred fir (*Abies religiosa*) is widely distributed within the Trans‐Mexican Volcanic Belt at elevations mostly between 2800 and 3500 m above sea level, though a few trees can be found as low as 2400 m (Sáenz‐Romero et al. 2024). *A. religiosa* is a valued timber species and a dominant tree species within its range but, as in many forests around the world, climate change is increasing sacred fir mortality due to hotter droughts and bark beetle outbreaks (Gómez‐Pineda et al. 2022; Hammond et al. 2022). By 2090, the area suitable for the *Abies religiosa* is likely to shrink 96% nationwide (Sáenz‐Romero et al. 2012). Sacred fir is an important species for conservation of the migratory monarch butterfly as well (*Danaus plexippus L*.) (Cruzado‐Vargas et al. 2021) as the dense crowns of *A. religiosa* provide a moderate temperature for butterflies under conditions where they would otherwise overheat or freeze (Anderson and Brower 1996). Unfortunately, one of the areas where the range of *A. religiosa* is projected to severely shrink due to climate change is the Monarch Butterfly Biosphere Reserve (Sáenz‐Romero et al. 2012). Better understanding the variation in climate responses within the species may help forest managers engaged in reforestation to select seeds that are likely to thrive in future conditions.

Although trees can migrate toward cooler areas through seed dispersal, long generation times, limited seed dispersal, and habitat fragmentation can limit the speed of tree migration (Aitken et al. 2008; Moran et al. 2022). This speed is likely to be lower than required to track anthropogenic climate change, even in many mountainous areas where the required dispersal distances are shorter (Lustenhouwer et al. 2017) as existing vegetation may limit establishment and, where shifts in elevation range have been observed, contractions of the lower range limit are more common (Kelly and Goulden 2008; Zu et al. 2021; Moran and Ormond 2015). Thus, human‐assisted migration of seed sources is increasingly considered a viable adaptive management strategy for many tree species, and tests are being carried out to make sure the benefits outweigh the risks (Carbajal‐Navarro et al. 2019; Young et al. 2020; Sáenz‐Romero et al. 2021). For *A. religiosa* specifically, when Cruzado‐Vargas et al. collected 11 provenances along an elevation gradient (3000 to 3550 m a.s.l.) and assayed these seedlings in three common gardens, they found that a 400 m shift upwards in elevation enhanced seedling performance (Cruzado‐Vargas et al. 2021).

Widespread tree species often show local adaptation to climate and associated genetic differences (McKenney et al. 1999; Gross et al. 2017). Understanding the genetic differences in climate response between various populations of trees plays an important role in selecting appropriate seed sources for reforestation. With the ability to analyze high numbers of SNP markers through methods such as Genotyping‐By‐Sequencing (GBS) (Andolfatto et al. 2011; C. Chen et al. 2013), genomic selection (GS) has become a feasible tree seeding selection strategy, which is projected to require less time than traditional selective breeding. Several studies have previously investigated functional variation involved in resistance to diseases (Torkamaneh et al. 2016), pests (Lebedev et al. 2020), and drought (Bouvet et al. 2020; Laverdière et al. 2022) by analyzing the functions of genes containing or adjacent to significant SNP markers. It has also been suggested that genomic data could be used to better target assisted migration efforts by, for example, using populations with higher frequencies of warm‐adapted alleles as sources and those with lower frequencies of warm‐adapted alleles as recipients (Chen et al. 2022).

Detection of between‐population differentiation in outlier loci (often based on F_ST_ methods) and genotype‐environment association (GEA) analysis are frequently used to identify genomic loci potentially associated with natural selection in forest trees (Yang et al. 2016; Martins et al. 2018). F_ST_ outlier methods identify loci with strong allele frequency differences among populations (Song et al. 2016), whereas GEA directly tests associations between allele frequencies and environmental variables, thereby linking genetic variation to specific environmental conditions (Meger et al. 2021; Cobo‐Simón et al. 2021). Among alternative GEA approaches, univariate models have shown strong performance and are widely used for detecting associations between individual loci and environmental variables (Forester et al. 2018). Several univariate GEA approaches, including Bayenv2 (Günther and Coop 2013), PCAdapt (Duforet‐Frebourg et al. 2014), and latent factor mixed models (LFMM) (Frichot et al. 2013), are widely used to identify such associations.

In 2019, Cruzado‐Vargas and Sáenz‐Romero established an assisted migration experiment in the Monarch Butterfly Biosphere Reserve where *Abies religiosa* seedling provenances from elevations of 3003 to 3552 m were planted into 3 common gardens at 2600, 3000, and 3400 m. By early 2022, > 84% of seedlings of all provenances were still alive in the middle garden, and > 90% of seedlings of all provenances were alive in the upper garden, but survival in the 2600 m garden ranged from 0% to 13.3% per provenance. While seedlings from lower elevation provenances tended to have higher survival at this hot range edge (see Figure 1 below), source elevation only weakly explained the pattern of survival. We therefore hypothesized that surviving seedlings might possess genetic variants linked to heat or drought tolerance that occur across elevations, but which may be more common at lower elevations.

**FIGURE 1:**
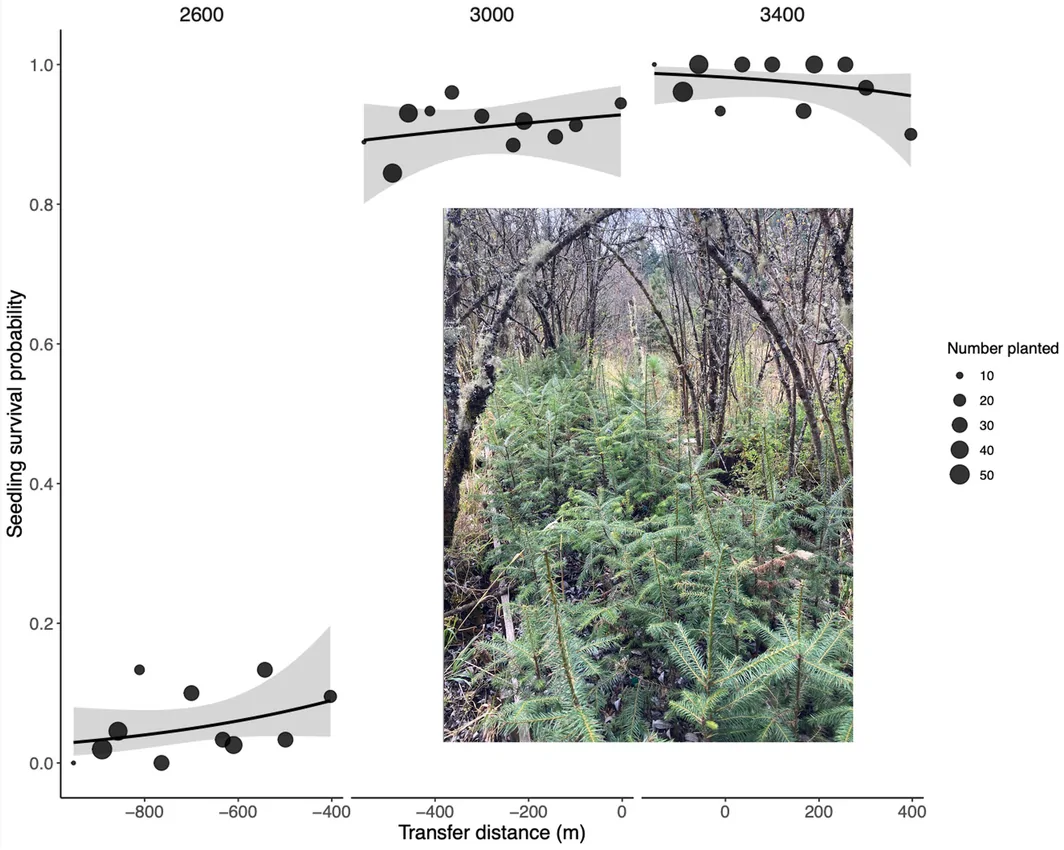
Graphs show *Abies religiosa* seedling survival at low, middle, and high elevation gardens relative to elevation transfer distance. Transfer distance was calculated as the elevation of the planting site minus the elevation of the provenance source. The inset photograph shows high elevation garden layout, 2023. (Photo by E. Moran).

In the analyses detailed below, we therefore:

1. Examined population structure and genetic differentiation for *Abies religiosa* seedling groups planted across different elevations.
2. Tested for genetic differentiation between the survivor group, represented by surviving seedlings from the low‐elevation garden, and the initial group, represented by surviving seedlings from the mid‐ and high‐elevation gardens, using a PCAdapt outlier test.
3. Conducted a GEA analysis to identify SNPs associated with variation in seed source climate and then examined the overlap between these SNPs and those identified in Step 2 as putatively under selection.

## 2 Materials and Methods

### 2.1 Sample Collection and DNA Extraction

The *Abies religiosa* planting experiment was conducted within the Monarch Butterfly Biosphere Reserve (MBBR), located in the states of México and Michoacán (Gómez‐Pineda et al. 2021). Elevation in the MBBR ranges from 2200 to 3640 m a.s.l., with annual average temperatures from 8°C to 22°C (Cruzado‐Vargas et al. 2021). *A. religiosa* seedlings from 11 source populations were planted in shaded raised beds with the same soil mix into three sites at 2600, 3000, and 3400 m. Each site had 330 seedlings, distributed in six blocks per site, with 30 seedlings per provenance per site.

Transfer distance was calculated as the elevation of the planting site minus the elevation of the provenance source, so that positive values indicate an upward transfer and negative values indicate a downward transfer. We first examined the relationship between elevation transfer distance and seedling survival. For the low elevation garden, we would expect low elevation populations with a less negative transfer distance to show higher survival than high elevation populations. The reverse would be expected for the high elevation garden, if any difference in survival were seen. Because survival differed substantially among gardens, we analyzed them separately, modeling the number of surviving and nonsurviving seedlings per provenance using generalized linear models (GLMs) with a binomial error distribution and logit link function in R (*glm*, family = “binomial”), with elevation transfer distance as the predictor.

Needle samples were collected for all surviving seedlings from the low‐elevation garden (*N* = 19) and 17–20 individuals per source population from the high‐ and mid‐elevation gardens (*N* = 216). Details of which seedlings survived in each raised bed and which were sampled are shown in Appendix S1. After collection, needle samples were dried over silica gel and shipped to UC Merced for DNA extraction. The 235 samples' genomic DNA was extracted by using the DNeasy Plant Pro Kit (QIAGEN, Hilden, Germany) according to the manufacturer's protocol (Urbina et al. 2022). Nanodrop was used to test the concentration of extracted DNA, which ranged from 76.0 to 338.1 ng/ul (Gattis and Reza 2023). Frozen DNA samples were shipped to the UC Davis Genomic Center for GBS. For subsequent analyses, we combined the samples from the high‐ and mid‐elevation gardens as the “initial” population because, with their high survival levels, they should represent the initial allele frequencies present in all the gardens, including the initial population from which low‐elevation survivors were selected.

The “initial” population was represented by 216 individuals from the high survival mid‐ and high‐elevation gardens, whereas only 19 surviving individuals were available from the low‐elevation range‐edge garden. Although this resulted in an unbalanced sample size between groups, we retained all individuals in the initial population to obtain the most precise estimates of allele frequencies and to avoid reducing the information available through random subsampling. The unequal sample sizes were considered when interpreting the genetic differentiation between the initial and survivor groups and are acknowledged as a limitation of the study.

### 2.2 GBS Library Preparation and DNA Sequencing

Two 96‐plex GBS libraries consisting of 192 DNA samples and a 48‐plex GBS library consisting of 43 DNA samples and a negative control (no DNA) were prepared in this study. Each of the samples in each library was tagged with a unique barcode. 100 ng DNA extracts were digested with the restriction enzyme *Ape*KI at 75°C for 2 h. Sequences for the *Ape*KI barcode adapters and the common adapters, and the temperature cycles were as described in Shu and Moran (2020). The fragments from all 236 samples were then sequenced on NovaSeqX, paired‐end 150 bp (Illumina, San Diego, CA).

### 2.3 De Novo Assembly and SNP Identification

Unlike *Pinus* (Neale et al. 2014, 2024; Stevens et al. 2016; Niu et al. 2022) and *Picea* (Gagalova et al. 2022), where multiple species per genus have been sequenced, allowing comparison of genome structure and sequence, the only genome sequence available for *Abies* is for the European silver fir, *Abies alba* . This sequence is still fragmentary, being composed of 37 million scaffolds of 106–297,427 bp in length, totaling 18.9 Gb, with a completeness estimate of 69% based on core gene comparison to *Pinus taeda* (Mosca et al. 2019). Moreover, *Abies religiosa* is most closely related to other warm‐climate Western North American firs, such as *A. concolor* or *A. grandis* , while *Abies alba* falls into a divergent clade that split off around 15–20 million years ago and includes most Eurasian species and the boreal North American firs *A. lasiocarpa* , *A. balsamea* , and *A. fraseri* (Semerikova and Semerikov 2014; Semerikova et al. 2018). However, while the broader clade to which *A. religiosa* belongs is one of the more basal, *A. religiosa* and its closest relatives (all Mesoamerican firs) are actually from a young and rather poorly differentiated group (Semerikova et al. 2018). Because of these factors, we decided to use a de novo approach to SNP calling for *A. religiosa* .

Because of its strong performance in a previous methods comparison (Shu and Moran 2020), assembly and SNP calling were performed using the STACKS‐2.59 *de novo* pipeline. Reads were demultiplexed, cleaned, and trimmed to 64 bp. The quality score limit was set to 20, the window size to 0.20, and other parameters with default settings (Shu and Moran 2020). To limit false SNP identification and increase the accuracy of downstream analyses, SNPs were filtered by using VCFtools. We selected only biallelic SNPs (min‐alleles 2, max‐alleles 2), where at least 25% of individuals were genotyped at that SNP (max‐missing 0.75) and the minor allele frequency was at least 5% (maf 0.05), minimum depth equal to half of the mean of the depth distribution (minDP 5), and Hardy–Weinberg Equilibrium value was 0.00001 (hwe 0.00001) (Nishio et al. 2022).

### 2.4 Population Structure and Genetic Differentiation Analyses

Principal component analysis (PCA) was conducted for all 18,816 SNPs using the function *glPCA* implemented in the R package *adegenet* (Matheson et al. 2023; Jiang et al. 2021). Population genetic differences were calculated using the *populations* program in STACKS‐2.59 for the initial and survivor groups (Wu et al. 2024). Observed heterozygosity, expected heterozygosity, observed homozygosity, expected homozygosity, an estimate of nucleotide diversity, and the inbreeding coefficient of an individual (I) relative to the subpopulation (S) were automatically calculated by STACKS (Catchen et al. 2011; Gladfelter et al. 2020). Initial and survivor groups were pairwise compared to compute the F_ST_ for each locus (Alonso‐García et al. 2021).

### 2.5 Outlier Detection for Biological Adaptation

We chose PCAdapt to test for signals of potential selection. The creators of this program showed that F_ST_ can be viewed as the proportion of variance in genotype that is explained by PCA, with the correlation between genetic variants and each PC providing a framework to detect variants involved in adaptation without pre‐defining populations, as is required by classic F_ST_ analysis (Duforet‐Frebourg et al. 2014). Compared to other computer programs for genome scans (BayeScan, hapflk, OutFLANK, sNMF), it was shown to have the lowest false discovery rate (FDR) when employed to identify outlier SNPs (Luu et al. 2017; Wang et al. 2023). The commonly used BayeScan also requires discrete populations, whereas our samples represent a before and after of the same group. PCAdapt identifies outlier SNPs with respect to population structure through PCA; SNPs deviating significantly from the neutral background structure along the principal components (*p*‐values < 0.05) were identified as outlier loci (Jombart et al. 2010). For this analysis, we chose *K* = 3 based on the population structure analysis result.

### 2.6 Genotype‐Environment Association

Average climate variables for the source populations (provenances) between 1961 and 1990 were estimated using climate spline models (https://charcoal2.cnre.vt.edu/climate/), based on Sáenz‐Romero et al. 2010 for the Mexican extent. Environmental variables were pruned prior to GEA analysis to reduce multicollinearity, which can affect the sensitivity of association models (Felkel et al. 2023). After calculating the correlations among the 20 available environment variables, five climate variables with pairwise correlation coefficients |r| < 0.9 and variance inflation factor (VIF) < 10 were selected for GEA by using functions vifstep and vifcor in the R package usdm. The vifcor function identifies highly correlated variable pairs, excluding those with higher VIF, and repeats this process until no strong correlations remain. Similarly, vifstep calculates VIF for all variables, removes the one with the highest VIF, and repeats the process until all remaining variables meet the VIF threshold (Khayat and Dakhil 2024; Daï et al. 2023). The selected climate variables were degree‐days < 0°C, summer precipitation (July–August), winter precipitation (November–February), Julian date of the last spring freeze, and summer precipitation balance (ratio of summer to spring precipitation). Other climate variables considered but not included in the analysis were mean annual temperature; mean annual precipitation; growing season precipitation (April–September); mean temperature in the coldest month; mean minimum temperature in the coldest month; mean temperature in the warmest month; mean maximum temperature in the warmest month; Julian date of the first freezing date of autumn; length of the frost‐free period; degree‐days > 5°C (based on mean monthly temperature); degree‐days > 5°C accumulating within the frost‐free period; Julian date the sum of degree‐days > 5°C reaches 100; degree‐days < 0°C (based on mean minimum monthly temperature); summer/spring precipitation balance ((July+August)/(April+May)); and spring precipitation (April+May).

Latent factor mixed model 2 (LFMM2) was used for GEA association due to its fast computing speed and effectiveness in controlling for demographic effects and population structure (Wang et al. 2017). Both PCA and admixture analysis, the two methods available in the LEA v.2.6.0 R package, were used to determine population structure K (Lazic et al. 2024). The LEA function snmf was run for *K* values between 1 and 11 with 10 repetitions each. The best K value was determined by minimizing cross‐validation error using a 10‐fold method. After conducting the GEA with LFMM2, raw *p*‐values were adjusted with the Genomic Inflation Factor (GIF) to correct for biases from population structure and other factors (Shu and Moran 2023). Results with a *p* (< 10^−5^) value were considered significant associations (De Kort et al. 2014; Abebe et al. 2015).

### 2.7 Selected SNPs Distribution and Classification

The categories of PCAdapt/LFMM‐selected SNPs were classified by type of point mutation. Transversions are more likely to affect amino acid sequence and the binding of transcription factors (thus affecting gene expression) than transitions (Guo et al. 2017). Transition mutations, on the other hand, tend to occur 2–4 times as frequently as transversions because they are between bases with the same ring structure (Aziz et al. 2022). Transitions tend to be observed even more frequently within coding regions where conservation of function is favored. While we cannot confidently identify synonymous vs. non‐synonymous mutations or gene functions without being able to align sequence fragments with an annotated genome sequence, the ratios of transitions to transversions might provide some information about the likely functional consequences of mutations. Patterns of nucleotide substitutions in SNPs (e.g., G to A transitions) may be associated with plant stress responses (Maghuly et al. 2018; Parida et al. 2012). Therefore, the mutation types of the detected SNPs were also classified.

The distribution and classification of selected SNPs as major or minor allele and the type of transition or transversion were summarized and compared using SCI‐VCF R version (Kamaraj and Sinha 2024). Venn diagrams, allele frequency histograms, and heatmaps were drawn using the BioLadder platform (Zhang et al. 2024).

### 2.8 Allele Frequencies for GEA Selected SNPs

Allele frequencies for GEA‐selected SNPs of total samples were extracted by VCFtools after quality control filtering. After extracting the genetic information of selected SNPs within the survivor group vs. the initial group, locus allele frequency (AF) for each locus was recalculated by using the formula:

$$\mathrm{AF}=\frac{\text{AN}}{\mathrm{AC}}$$

where AC (Allele Count) represents the total occurrences of a particular allele in the population, and AN (Allele Number) is the total number of alleles observed at that locus.

## 3 Results

### 3.1 Transfer Distance Analysis

Elevation transfer distance was not significantly associated with seedling survival at any of the three planting elevations (Figure 1). At the low‐elevation garden (2600 m), survival showed a positive, although nonsignificant, relationship with transfer distance (GLM, *p* = 0.180), providing limited evidence that populations originating from lower elevations may have performed better under the warmer conditions at this site. No significant relationship between transfer distance and survival was detected at the 3000 m garden (*p* = 0.537) or the 3400 m garden (*p* = 0.311). Thus, while transfer to lower elevations significantly reduced survival, elevation transfer distance did not significantly predict survival within a given garden.

The provenance survival differences among common gardens were not explained by elevation transfer distance alone. This suggests that the relationship between provenance origin and performance may involve more complex climatic, environmental, or genetic differences than can be captured by a simple measure of elevational displacement. Genomic analyses were therefore used to investigate whether particular loci showed patterns consistent with environmental adaptation or population differentiation.

### 3.2 SNP Discovery

A total of 1,511,227 variant sites were identified from the GBS data for the 236 seedlings. 346,114 SNPs in the “initial” group and 38,509 SNPs of the “survivor” group were found to be significantly out of Hardy–Weinberg equilibrium (< 0.05). After the quality and depth filtering by VCFtools, 98.76% of loci were removed and 18,816 high‐quality SNPs were retained. Of these filtered SNPs, 11,780 (62.61%) were classified as transitions and 7036 (37.39%) were classified as transversions. Distributions of each type of SNP were as follows: C/T, 5937 (32.29%); A/G, 5843 (31.05%); A/C, 1901 (10.10%); G/T, 1867 (9.92%); C/G, 1736 (9.23%); and A/T, 1532 (8.14%), as shown in Figure 2.

**FIGURE 2:**
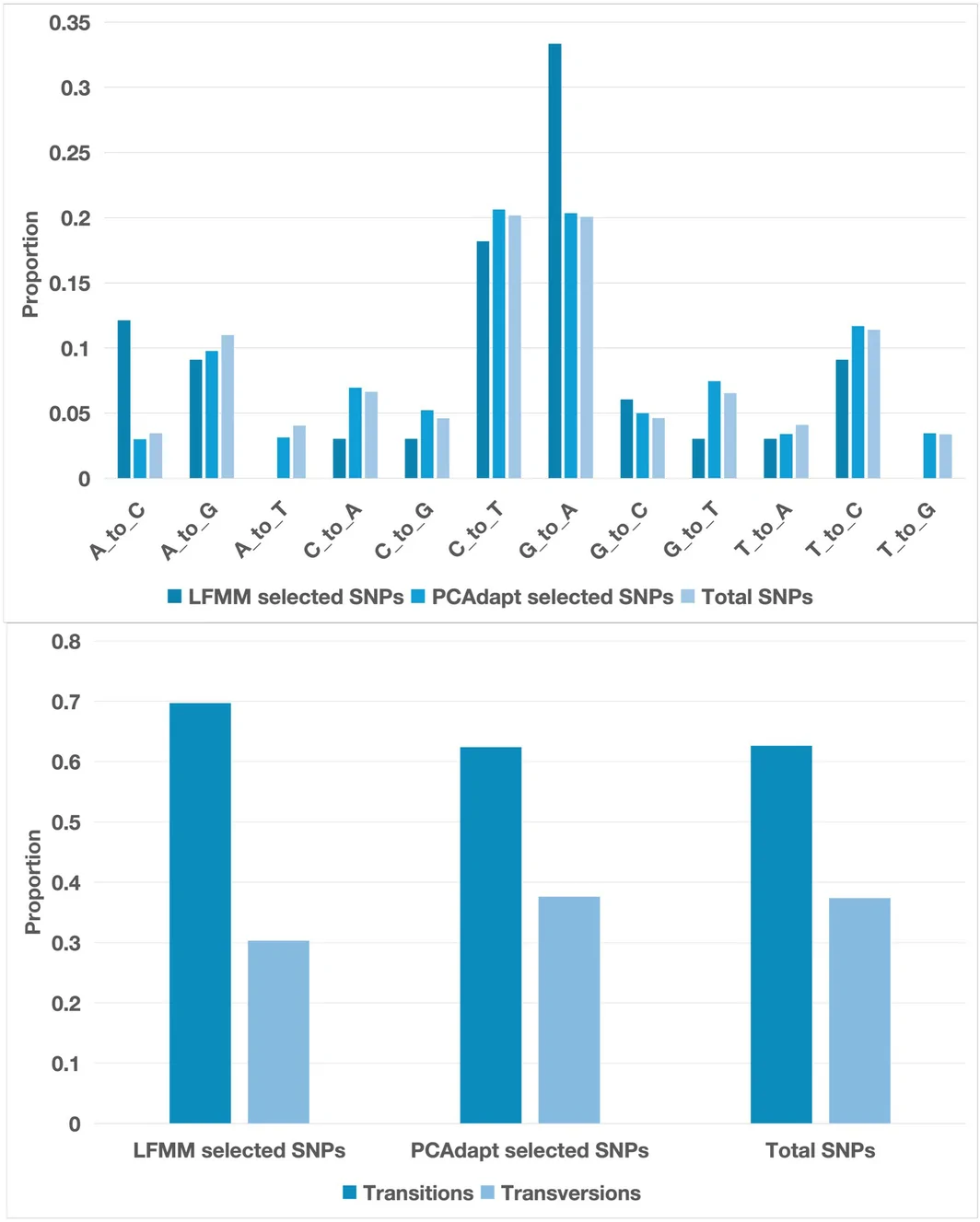
Distribution of SNP type for total SNPs versus outliers detected by PCAdapt or LFMM (top); Distribution of transitions vs. transversions for total SNPs versus outliers detected by PCAdapt or LFMM (bottom).

### 3.3 Population Structure and Genetic Differentiation

The F_ST_ value between the initial group and survivor group is 0.00256. Appendix S2 shows the genetic difference result for the initial and survivor groups. The first three PC axes explained 3.02% (PC1), 1.92% (PC2), and 1.85% (PC3) of the total genetic variation (Figure 2); these low percentages are typical of analyses using thousands of SNPs (Goebl et al. 2024; Gauch et al. 2019). Some provenances are more strongly differentiated from one another, while others cluster together. Initial and survivor populations, on the other hand, are not strongly differentiated when looking at all SNP data together. This is to be expected, given that survivors are essentially a subset of the initial and would not be expected to differ across all genetic loci.

### 3.4 SNP Outlier Detection by PCAdapt

We identified 2202 outlier SNPs, and of these 1374 (62.40%) were classified as transitions and 828 (37.60%) were classified as transversions. Distributions of each type of SNP were as follows: A/G, 663 (30.11%); C/T, 711 (32.29%); A/T, 144 (6.54%); A/C, 219 (9.95%); C/G, 225 (10.22%); and G/T, 240 (10.90%). SNP type distribution was shown for adapted SNPs identified by PCAdapt (Figure 2). These proportions are similar to those for all filtered SNPs, with A/G and C/T mutations (transitions) making up nearly a third of the total each.

### 3.5 SNPs Associated With Environmental Variables

Based on the results of admixture analysis in the LEA package (Figure 3), cross‐entropy decreased sharply at *K* = 6 and the lowest cross‐entropy occurred at K = 11. K = 6 was considered to represent the main genetic structure while *K* = 11 suggested additional fine‐scale population subdivision. The PCA result indicated similar clusters. Therefore, both *K* = 6 and *K* = 11 were examined to represent major and finer genetic structure, respectively. Twenty‐nine GEA loci were identified at *K* = 6, while 31 were identified at *K* = 11. Summarizing the selected loci by *K* = 6 and *K* = 11, 33 SNPs were found to be associated with climate variables; 23 (69.70%) were classified as transitions, and 10 (30.30%) were classified as transversions. This is a slightly higher ratio of transitions compared to total SNPs or the SNPs identified by PCAdapt. This was driven by a particularly large proportion of A/G transitions (42.42%) of which 11/14 are G to A, while the proportion of C/T transitions was somewhat lower (27.27%), of which 6/9 are C to T. The remaining mutation types were A/T, 1 (3.03%); A/C, 5 (15.15%); C/G, 3 (9.09%); and G/T, 1 (3.03%) (Figure 2).

**FIGURE 3:**
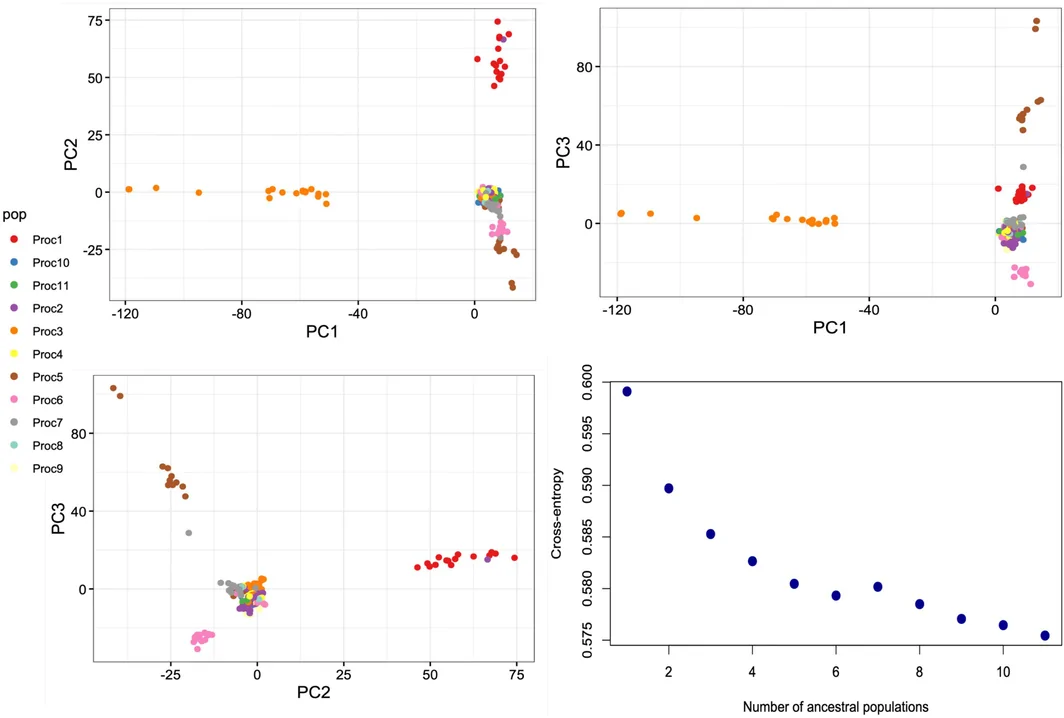
PCA analysis results (top left, top right, bottom left) and admixture analysis result (bottom right) for LEA package. In PCA analysis figures, the colors of the dots indicate which of the 11 different provenances the seedling in question came from, axis values correspond to PCA scores for each individual. In the admixture analysis figure, the x‐axis shows the number of proposed populations, and the y axis is the cross‐entropy.

Out of 33 climate‐associated SNPs, 1 was associated with all five selected climate variables, 6 were associated with more than one of the selected climate variables, and 26 were found to be associated with only one of the variables. SNPs associated with summer precipitation, Julian date of the last freezing date of spring, and summer precipitation balance were the ones most often associated with multiple climate variables. Table 1 shows the classification for all climate‐associated SNPs. Venn diagrams (Figure 4) showed the overlapping for SNPs associated with different kinds of climate variables. These variables were degree‐days < 0°C; summer precipitation (July–August); winter precipitation (November–February); Julian date of the last spring freeze; and summer precipitation balance.

**TABLE 1:** Classification for SNPs associated with climate variables.

Climate variables	Total SNPs	Unique SNPs
dd0 (degree‐days < 0°C)	11	7
Sday (julian date of the last spring freeze)	11	4
Smrp (summer precipitation)	9	1
Smrpb (summer precipitation balance)	2	0
Winp (winter precipitation)	15	14
Total unique snps from *K* = 6 and *K* = 11	33	

**TABLE 2:** Location and climate average for the 11 provenances (1961–1990, from https://charcoal2.cnre.vt.edu/climate/, based on Sáenz‐Romero et al. 2010) and the three planting sites (from data loggers and rain traps during the experiment: 2019–2021).

Provenance	Elev. (m)	Lat. N	Long. W	MAT	MAP	dd0	Sday	Smrp	Winp	Smrpb
1	3552	19.564	100.229	8.5	1107	6	96	416	90	1.94
2	3491	19.567	100.233	8.8	1094	4	97	412	89	1.94
3	3457	19.571	100.235	9.0	1089	3	97	411	89	1.95
4	3411	19.573	100.237	9.3	1079	1	96	408	88	1.96
5	3364	19.575	100.234	9.5	1065	1	92	405	88	1.98
6	3300	19.579	100.231	9.9	1048	0	88	399	87	1.99
7	3233	19.580	100.224	10.2	1029	0	85	393	87	2.00
8	3210	19.581	100.220	10.3	1022	0	84	390	87	2.01
9	3143	19.581	100.214	10.6	1001	0	84	384	86	2.03
10	3099	19.586	100.214	10.8	987	0	84	381	85	2.04
11	3003	19.595	100.210	11.3	969	0	87	388	76	2.13
High garden	3400	19.57	100.23	6.9	1270	—	—	507	105	2.10
Mid garden	3000	19.58	100.18	12.8	1040	—	—	488	56	3.18
Low garden	2600	19.80	100.16	14.6	895	—	—	429	26	3.69

Abbreviations: dd0, degree‐days < 0°C; MAP, Mean annual precipitation (mm); MAT, Mean annual temperature (°C); Sday, Julian date of the last spring freeze (days); smrp, summer precipitation (July–August; mm); smrpb, summer precipitation balance (ratio of summer to spring precipitation); winp, winter precipitation (November–February; mm).

**FIGURE 4:**
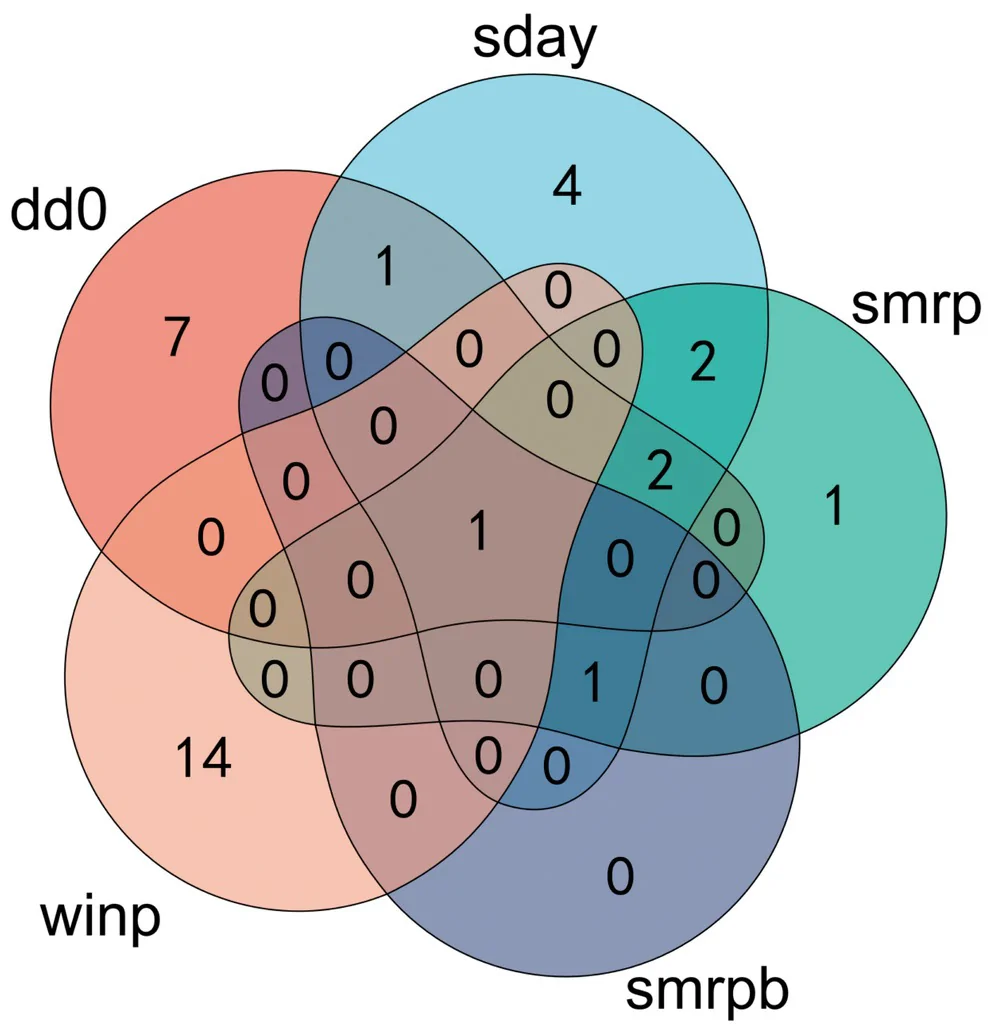
Overlapping of 33 climate associated SNPs for five selected climate variables. Variables include dd0‐degree‐days < 0°C, smrp‐summer precipitation (July–August), winp‐winter precipitation (November–February), sday‐julian date of the last spring freeze and smrpb‐summer precipitation balance. Each color represents a climate variable, and the number indicates the count of SNPs associated with that variable. Overlapping areas show the number of SNPs associated with more than one climate variable.

### 3.6 Genetic Differences Between Survivor and Initial Seedling Groups, as Identified by Various Methods

Of the 2202 SNPs identified by PCAdapt as potentially under selection, 4 overlapped with the 33 SNPs identified by the GEA analysis (Figure 5a). Of these 4 overlapping SNPs, 3 were associated with winter precipitation while 1 of them was associated with degree‐days < 0°C. Based on Figure 2, LFMM‐selected SNPs tended to have a higher rate for “G to A” and “A to C” with a lower rate of “A to T” and “T to G” compared to total SNPs and PCAdapt‐selected SNPs.

**FIGURE 5:**
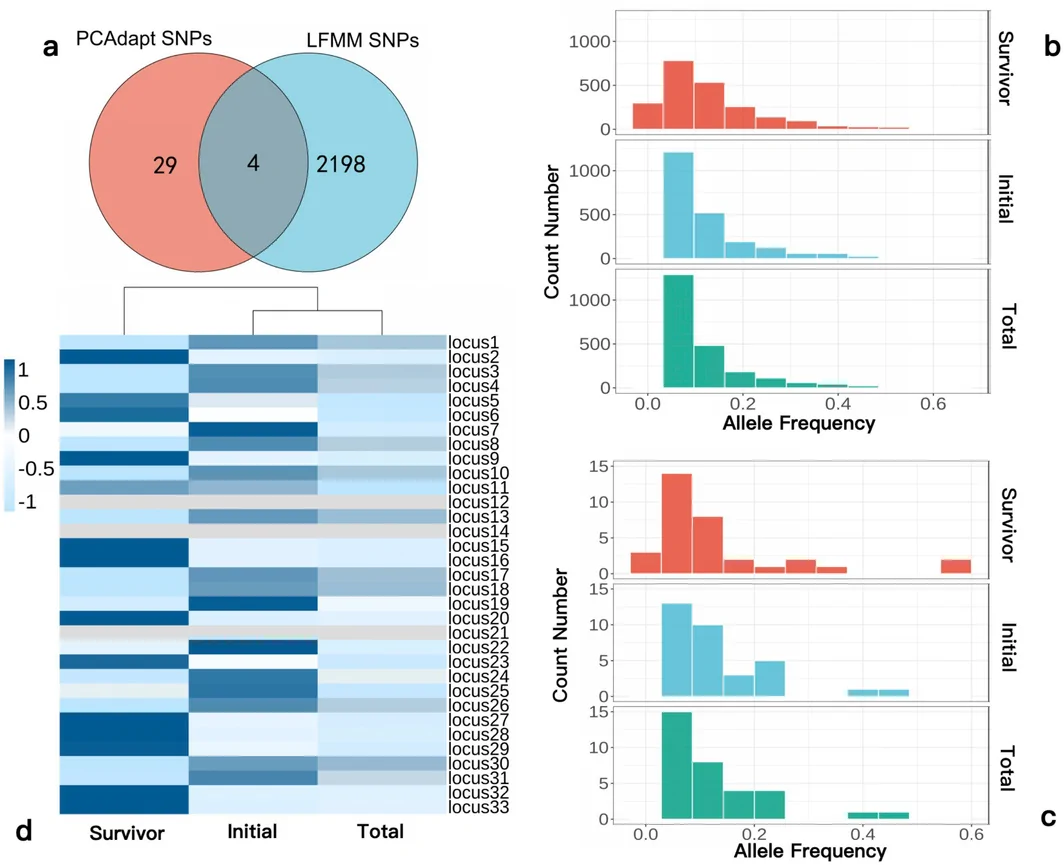
Overlapping between SNPs selected by PCAdapt and SNPs selected by the LFMM model (a). Minor allele frequency distribution for SNPs selected by PCAdapt for different groups (b). Minor allele frequency distribution for SNPs selected by the LFMM model for different groups (c). Heatmap with clustering based on the Euclidean distances for 33 LFMM‐selected SNPs frequency (d).

Figure 5b–d show SNP differences within initial and survivor seedling groups. Minor allele frequencies of the 2022 SNPs identified by PCAdapt as potentially under selection are shown in Figure 5b. The mean and standard deviation for the initial group, the survivor group, and all seedlings together were 0.124 ± 0.089, 0.127 ± 0.104, and 0.118 ± 0.087. For the 33 SNPs selected by LFMM, minor allele frequency means and standard deviation for the initial group, the survivor group, and all seedlings together were 0.137 ± 0.100, 0.133 ± 0.139, and 0.127 ± 0.099. Thus, we can see that the survivor group in both cases tends to exhibit more variation in minor allele frequency than the initial group.

We also conducted an F‐test for equality of variances for the initial group and survivor group based on 33 LFMM‐related loci; there's no statistically significant difference between the two groups. However, based on Cohen's test, the cumulative differentiation across multiple loci suggests potential for adaptive seedling sourcing even if individual locus differences are subtle. A few loci (e.g., locus20, locus33, locus21) approach moderate effect sizes (0.4–0.46) (Appendix S3).

## 4 Discussion

### 4.1 Population Genetic Structure

Genetic variation in many tree species can be affected by broad dispersal of their pollen (Dick et al. 2008; Ndiade‐Bourobou et al. 2010; Gerber et al. 2014). Unlike seeds, which are heavier and have more restricted dispersal, pollen can travel long distances, which causes genetic differences between neighboring areas to blur when looking at genome‐wide data (Omondi et al. 2023; Gerber et al. 2014). However, while overall genetic differentiation between *A. religiosa* populations is low, we can also see clustering of genotypes by source provenance (Figure 6), indicating that gene flow has not fully homogenized them.

**FIGURE 6:**
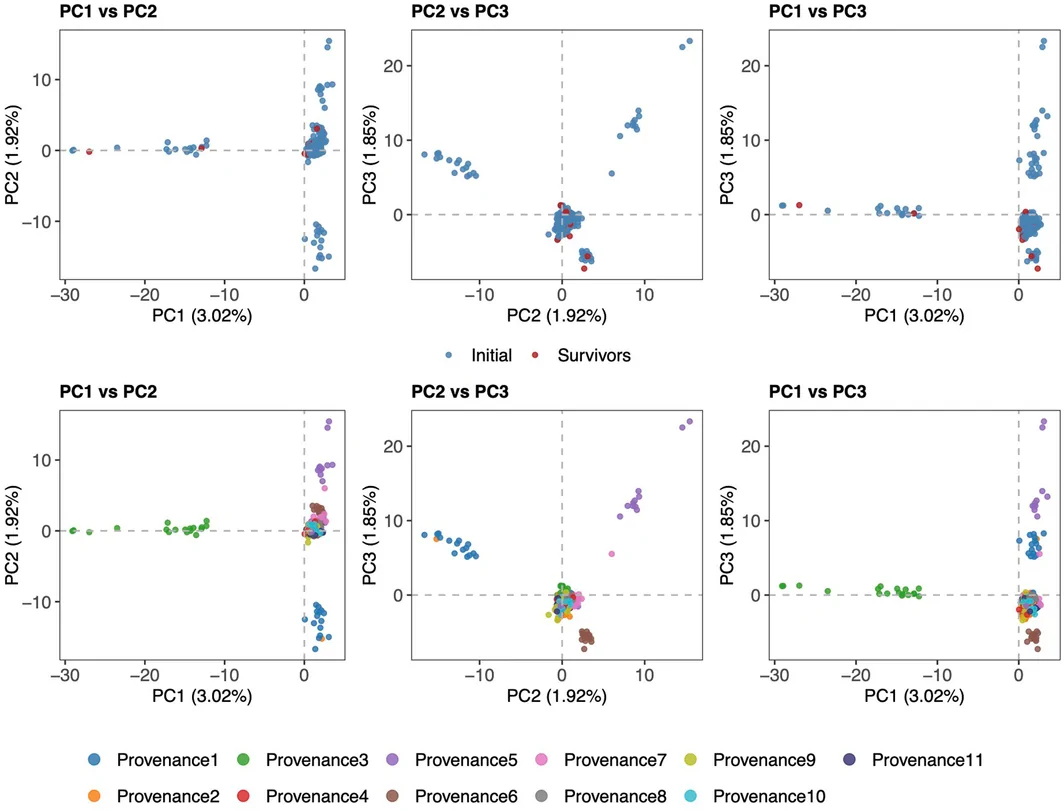
PCA result of *Abies religiosa* SNP data. The top three figures show the results for samples as initial planting group (1; blue) vs. survivor group (2; red). The bottom three figures show the results with samples classified by provenance, as listed in Table 2. The most differentiated provenances were 1 (3552 m), 3 (3457 m), 5 (3364 m), and 6 (3300 m).

The results of this study support the idea that PCAdapt and GEA analyses can identify loci potentially under selection even between groups with low genetic differentiation (Yuan et al. 2023; Méndez‐Cea et al. 2023). Based on Figure 2 and Appendix S2, when considering all high‐quality genetic variant data, the genetic difference between the initial group and the survivor group was low (F_ST_ 0.00256). However, when comparing the allele frequency of adaptive SNPs (PCAdapt) and climate‐associated SNPs (GEA analysis) between the initial group and the survivor group, different patterns emerged. The minor allele frequency range in the survivor group tended to be wider for SNPs selected by PCAdapt and LFMM than for the initial group (or for the total sample group sample, which was numerically dominated by initial seedlings). Where the minor allele has a functional impact on seedlings, a novel or stressful environment could result in selection that drives its frequency either up or down in survivors, which could result in this pattern of more variable minor allele frequencies than in the initial population. These changes in frequency can be seen on a locus‐by‐locus basis in Figure 5d.

### 4.2 Adaptation to Climate

Habitat suitability for *A. religiosa* is significantly influenced by the mean temperature of the warmest month and the interplay between summer precipitation and winter temperatures (Sáenz‐Romero et al. 2012, 2024). This species is also vulnerable to extreme conditions, such as freezing temperatures and hot, dry spells, which make the presence of nurse shrubs essential for successful establishment (Carbajal‐Navarro et al. 2019). Given these sensitivities, it makes sense that summer precipitation, winter precipitation, and summer precipitation balance, degree days below 0°C (dd0), and the Julian date of the last freezing date of spring (sday) were identified as being associated with genetic variation. However, climate variables are often correlated (e.g., temperature and precipitation may both vary seasonally). This multicollinearity can distort the results, making it difficult to identify the specific contribution of each variable to observed genetic patterns (Nadeau et al. 2016). However, while our analysis only eliminated climate variables with |r| > 0.9, we still found very little overlap in SNPs being associated with different climate variables. Thus, the high proportion of SNPs found to be significant in the GEA being associated with winter precipitation may be due to a real relationship, especially because winter is the season with overall lower precipitation in this region.

Only 4 of the 33 SNPs identified by the GEA analysis overlapped with the 2202 identified by PCAdapt as potentially under selection, but this is not too surprising given the differences between the two approaches. PCAdapt aims to identify loci that show differences between populations, accounting for population structure but not environmental variation, whereas LFMM identifies associations between genetic variation and specific environmental variables. However, the association of 3 out of 4 of the overlapping SNPs with the amount of winter precipitation suggests that this climate factor is worth further investigation as a potential selective factor shaping these populations.

### 4.3 Minor Allele Frequency Variation

LFMM detected a smaller number of SNPs as significantly associated with climate gradients than PCAdapt identified as differing between populations. This difference likely reflects the fact that LFMM specifically tests for associations with environmental variables, whereas PCAdapt detects loci that may be influenced by multiple factors beyond climate. The standard deviation of minor allele frequencies in LFMM‐identified SNPs is higher compared to PCAdapt‐identified SNPs (Figure 5b,c). This could be due simply to sampling effects–as the former has a sample size of 33 SNPs and the latter 2202–but the change in allele frequencies between the initial and survivor groups is quite pronounced for many of the climate‐associated SNPs. Future studies could test whether indeed populations with higher frequencies of alleles associated with lower winter precipitation do generally perform better in winter‐dry sites, for example.

### 4.4 SNP Type

The ratios of transitions/transversions were 1.67 in the total SNP dataset, 1.66 in those identified by PCAdapt, and 2.3 in those identified by LFMM. This is consistent with the broad pattern of transitions occurring more often and transversions more frequently being selected against in coding or regulatory regions due to the higher likelihood of them affecting gene function (Guo et al. 2017; Aziz et al. 2022). For example, the overall transition to transversion ratio is 1.77 in peach trees, 1.56 in grapes, and 1.27 in apple trees (Aranzana et al. 2012). While the frequency of different types of mutations did not differ significantly between our three sets of SNPs, we observed a trend toward a higher frequency of A/G transitions and especially G‐to‐A mutations in the LFMM SNP data. Variation in the amount of GC bases can affect genome function, because GC pairs are held together by three hydrogen bonds, while AT pairs are connected by two. Some studies of noncoding regions in mammals suggest that G/C‐to‐A/T changes may occur more frequently than the reverse, with G‐to‐A mutations (or C‐to‐T, on the other strand) also being the most common type in the human genome (Jiang and Zhao 2006).

### 4.5 Limitations

The genetic data of this study had a high error rate and high missing data rate, which is typical for GBS and de novo SNP calling (Torkamaneh et al. 2016; Liao et al. 2019). Linkage disequilibrium (LD) tends to be quite low in conifers, so large gaps between SNPs could result in some of the variation responsible for local adaptation being missed (González‐Martínez et al. 2006). Additionally, the sampling locations were not representative of the entire range of *Abies religiosa*, and thus likely do not capture the full spectrum of environmental conditions experienced by the species and populations' genetic adaptations to it. This is a consequence of the gardens initially being established for a project with different goals (to test the viability of assisted migration of *Abies religiosa* provenances to higher elevations to compensate for climatic change, inside the Monarch Butterfly Biosphere Reserve). A recent study indicates that this species may show genetic variation in response to soil edaphic conditions (Arenas et al. 2025), so this should also be taken into account along with climate in future studies. Moreover, the low numbers of survivors made it difficult to determine if differences in the distribution of SNP types are biologically or statistically significant. In addition, alternative explanations, such as family effects or relatedness among individuals, could also contribute to the observed patterns. Lastly, LFMM models are primarily designed to detect linear associations between environmental variables and genetic variation, while trees might incorporate nonlinear responses to climatic extremes, including droughts (Caye et al. 2019).

### 4.6 Future Research

Conifer genomes are complicated to sequence and assemble due to their large size (18–35 Gb), but firs (*Abies*) are important components of many coniferous forests and hopefully more genome sequences for this genus will soon be made available as for their more distant pine (*Pinus*) and spruce (*Picea*) relatives. Two recent studies have used the *Abies alba* sequence to help identify SNPs in other fir species (Ousmael et al. 2023; del Valle et al. 2025); however, *A. nordmannia*, *Abies nebrodensis*, and *A. cephalonica* are all within the same European fir clade as *A. alba* , and thus much more closely related than *A. religiosa* . As the Ousmael et al. note, there are errors and uncertainties that could come both from aligning to a related‐but‐different species' genome, as well as errors and uncertainties that could come from assemblies made with low coverage sequencing, and indeed their reference‐free approach, Ebwt2InDel (Prezza et al. 2020), detected 95.9 million SNPs not identified by either reference‐based method. While we did not have the funding to do so, we recommend that future studies of *A. religiosa* or other species in its Western North America clade take a similar approach to Ousmael et al. if possible, sequencing of one or more megagametophytes to allow for better de‐novo SNP identification while testing the extent to which species from this divergent clade can align with the available *A. alba* genome. Sequence capture approaches based on the transcriptome can be helpful in identifying potential functional variation for species without a full genome sequence (Suren et al. 2016). If SNPs can be identified using an annotated genome from a related species, gene ontology (GO) and KEGG pathway mapping (Kanehisa and Goto 2000; Ashburner et al. 2000) would allow researchers to identify the putative function of nearby genes, including those that may be known to be involved in environmental stress response (Shu and Moran 2023).

SNPs in genes involved in stress responses, such as those controlling drought tolerance, pathogen resistance, or antioxidant defense, may exhibit particular patterns of nucleotide substitutions (e.g., G to A changes) (Dossa et al. 2019; Herwibawa et al. 2024; Hu et al. 2024). For example, nucleotide transitions like G to A can affect gene expression or protein function, influencing how plants react to stress, which might alter regulatory responses or modifications in biochemical pathways critical for stress tolerance (Assenov et al. 2013; Alonso et al. 2024). While more research is needed to definitively link SNP distributions like G to A to stress resilience across various species, there is potential for this approach to be used in predictive breeding models aimed at improving tree performance under environmental stress.

In both this case and for SNPs identified through GEA, a planting experiment would help in testing which SNPs are actually helpful for predicting performance in the field. For instance, a recent study found that the ability to predict growth and physiological traits in *Abies religiosa* based on SNPs identified in seedlings grown on two sites was low to moderate and did not transfer well across sites, indicating that the sample did not capture GXE interactions (Arenas et al. 2021).

## 5 Conclusion

Our analysis revealed subtle signatures of selection in *Abies religiosa* seedlings exposed to strong heat and drought stress. We also identified 33 SNPs associated with climate variation in source populations, particularly winter precipitation, and confirmed that minor allele frequencies were more variable in low‐elevation survivors for both these SNPs and those identified by PCAdapt, suggesting different alleles were favored relative to the initial population. As in previous studies, seedlings planted within the range performed somewhat better when transferred to higher elevations, while low‐elevation provenances performed at least somewhat better than high‐elevation provenances when planted below the range edge. Collectively, this suggests that there is existing genetic variation within *A. religiosa* populations that, through a combination of assisted migration and natural selection, could aid in the survival of this species under climate change. However, further work—particularly the development of genomic or transcriptomic resources—is needed if we are to better understand why certain genetic variants are associated with either long‐term climate variation or recent selection under hot, dry conditions.

## Author Contributions

**Yinyue Du:** data curation (equal), formal analysis (equal), investigation (equal), methodology (equal), visualization (equal), writing – original draft (lead), writing – review and editing (equal). **Emily V. Moran:** conceptualization (equal), data curation (equal), formal analysis (equal), funding acquisition (lead), methodology (equal), project administration (lead), resources (equal), supervision (equal), writing – review and editing (equal). **Ruli Zhang:** formal analysis (equal), visualization (equal). **Ana Laura Cruzado‐Vargas:** investigation (equal), writing – review and editing (supporting). **Cuauhtémoc Sáenz‐Romero:** investigation (equal), resources (equal), supervision (equal), writing – review and editing (equal).

## Conflicts of Interest

The authors declare no conflicts of interest.

## Supporting information


**Appendix S1:** Garden and needle collection maps.
**Appendix S2:** Genetic difference for initial group and survivor group. From left to right, each category represents observed heterozygosity, observed homozygosity, heterozygosity expected under Hardy–Weinberg equilibrium, homozygosity expected under Hardy–Weinberg equilibrium, an estimate of nucleotide diversity, the inbreeding coefficient of an individual (I) relative to the subpopulation (S).
**Appendix S3:** Cohen's D test: Cohen's D specifically measures the effect size of the difference between two means. Negative vs. positive signs just indicate direction of difference. Positive value means survivor group has higher values (allele frequencies) than initial group. Negative value means initial group has higher values than survivor group.

## Data Availability

The original raw sequencing data could not be recovered due to an irreversible hardware failure. However, the STACKS files, including haplotype sequences, consensus sequences for each locus, SNP calling output, and population genetics calculations, as well as excel files containing population source, DNA concentration, and barcodes for each sample, have been archived in Dryad (DOI: 10.5061/dryad.8gtht7726). All other downstream processed data (VCF files, analysis scripts, and intermediate outputs) have been deposited in Zenodo (DOI: 10.5281/zenodo.17717134).
