# Supplementary material for: Do Abies religiosa Seedlings That Survived Hot/Dry Range‐Edge Conditions Exhibit Genetic Evidence of Selection?
Source: Ecol Evol. 2026 Oct 20;16(10):e74455. doi: 10.1002/ece3.74455 (PMC13682914; doi:10.1002/ece3.74455)
Supplement: Supplementary file 1 [file ECE3-16-e74455-s001.docx]

Appendix 1: Garden and needle collection maps

Numbers in squares indicate which provenance a seedling came from. Dark grey squares indicate dead seedlings (where “-t” indicate a seedling killed by gophers). Blue squares indicate seedlings which we collected needles from, while white squares indicate live seedlings from which needles were not collected. PS = Seedlings taken randomly from provenances, to conform a edge row, to control border effects at the heads of the common gardens.

Appendix 2: Genetic difference for initial group and survivor group. From left to right, each category represents observed heterozygosity, observed homozygosity, heterozygosity expected under Hardy-Weinberg equilibrium, homozygosity expected under Hardy-Weinberg equilibrium, an estimate of nucleotide diversity, the inbreeding coefficient of an individual (I) relative to the subpopulation (S).

|  | Obs_Het | Obs_Hom | Exp_ Het | Exp_Hom | Pi | Fis |
| --- | --- | --- | --- | --- | --- | --- |
| Initial group | 0.23049 | 0.76951 | 0.23004 | 0.76996 | 0.23068 | -0.00137 |
| Survivor group | 0.23061 | 0.76939 | 0.22293 | 0.77707 | 0.2302 | -0.00394 |

Appendix 3: Cohen's D test : Cohen’s D specifically measures the effect size of the difference between two means. Negative vs positive signs just indicate direction of difference. Positive value means survivor group has higher values (allele frequencies) than initial group. Negative value means initial group has higher values than survivor group.

|  | effect size |
| --- | --- |
| locus1 | -0.409287765 |
| locus2 | 0.224586520225883 |
| locus3 | -0.172487017 |
| locus4 | -0.112195694 |
| locus5 | -0.003934551 |
| locus6 | 0.0112172855269769 |
| locus7 | -0.059760616 |
| locus8 | -0.124648252 |
| locus9 | 0.125726260928104 |
| locus10 | -0.233585152 |
| locus11 | -0.344023021 |
| locus12 | -0.4260684 |
| locus13 | -0.348262974 |
| locus14 | -0.295740532 |
| locus15 | 0.2743537262599 |
| locus16 | 0.220134867458986 |
| locus17 | -0.358227155 |
| locus18 | -0.152557285 |
| locus19 | -0.035509164 |
| locus20 | 0.463830017393216 |
| locus21 | -0.400318019 |
| locus22 | 0.0269462570513044 |
| locus23 | 0.060395657512386 |
| locus24 | -0.160163121 |
| locus25 | -0.091274505 |
| locus26 | -0.144270994 |
| locus27 | 0.0383414982096428 |
| locus28 | 0.283437484018695 |
| locus29 | 0.194933051669113 |
| locus30 | -0.248004406 |
| locus31 | -0.2600736 |
| locus32 | 0.211249688378156 |
| locus33 | 0.367762998200189 |
